# Supplementary material for: The interactome of intact mitochondria by cross-linking mass spectrometry provides evidence for coexisting respiratory supercomplexes
Source: Mol Cell Proteomics. 2017 Dec 8;17(2):216–32. doi: 10.1074/mcp.RA117.000470 (PMC5795388; doi:10.1074/mcp.RA117.000470)
Supplement: Supplemental Data [file supp_RA117.000470_133922_0_supp_23978_fzffwf.zip › spectra_annotation/mito_DR_spectra_annotation/473-1-4-1-3-1.pdf]

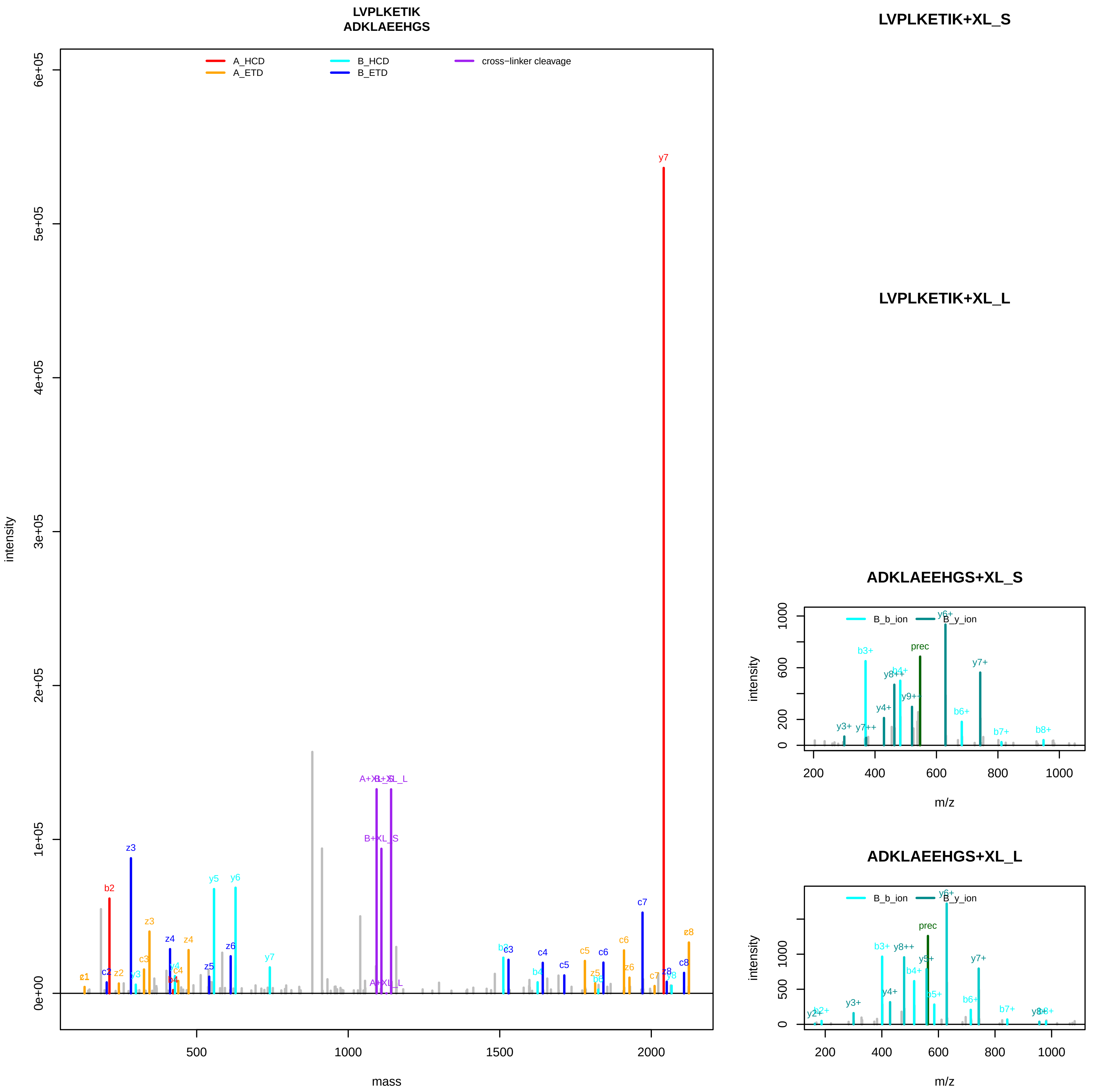

LVPLKETIK
ADKLAEEHGS
A_HCD
A_ETD
B_HCD
B_ETD
cross−linker cleavage
intensity
mass
0e+00
1e+05
2e+05
3e+05
4e+05
5e+05
6e+05
500
1000
1500
2000
y7
b2
z3
y5
y6
z4
z6
c3
z5
c2
z2
y3
c4
z1
c1
c7
c8
z8
y8
c6
c5
z7
c6
z6
c7
y7
b3
c4
c5
b4
A+XL_L
B+XL_S
LVPLKETIK+XL_S
LVPLKETIK+XL_L
ADKLAEEHGS+XL_S
B_b_ion
B_y_ion
intensity
m/z
0
200
600
1000
200
400
600
800
1000
y6+
b3+
prec
y7+
y8++
b4+
y9++
y4+
y3+
y7++
b6+
b7+
b8+
ADKLAEEHGS+XL_L
B_b_ion
B_y_ion
intensity
m/z
0
500
1000
200
400
600
800
1000
y6+
prec
b3+
y8++
y5+
b4+
y4+
y3+
b5+
b6+
y2+
b2+
b7+
